# Supplementary material for: Small Diameter Cell-Free Tissue-Engineered Vascular Grafts: Biomaterials and Manufacture Techniques to Reach Suitable Mechanical Properties
Source: Polymers (Basel). 2022 Aug 23;14(17):3440. doi: 10.3390/polym14173440 (PMC9460130; doi:10.3390/polym14173440)
Supplement: Supplementary file 1 [file polymers-14-03440-s001.zip › polymers-1775288-supplementary.pdf]

Review

Small diameter cell-free Tissue Engineered Vascular Grafts: Biomaterials and manufacture techniques to reach suitable mechanical properties.

María A. Rodríguez-Soto [1*], Camilo Andrés Polanía [2,4], Andrés M. Aragón-Rivera [1] Daniel Buitrago [1], María Ayala [1], Alejandro Velandia-Sánchez [2,4], Gabriela Peralta [1], Juan C. Cruz [1], Carolina Muñoz Camargo [1], Juan Guillermo Barrera-Carvajal [2], Jaime Camacho-Mackenzie [2] Juan Carlos Briceño [1,3,] *

Supplementary methodology

Figure S1. PRISMA algorithm for pre-clinical trials.

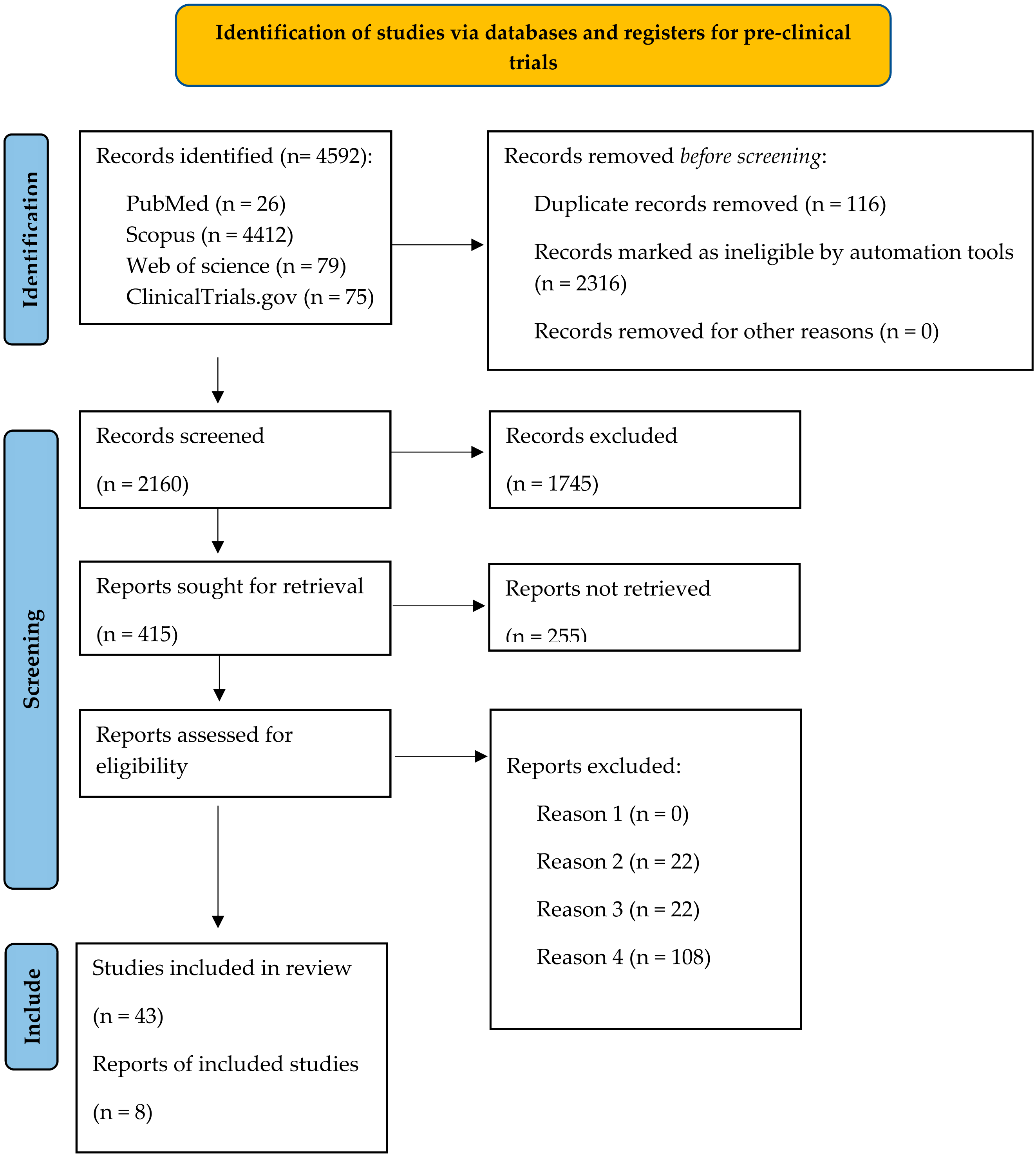

Figure S2. PRISMA algorithm for in vitro trials.

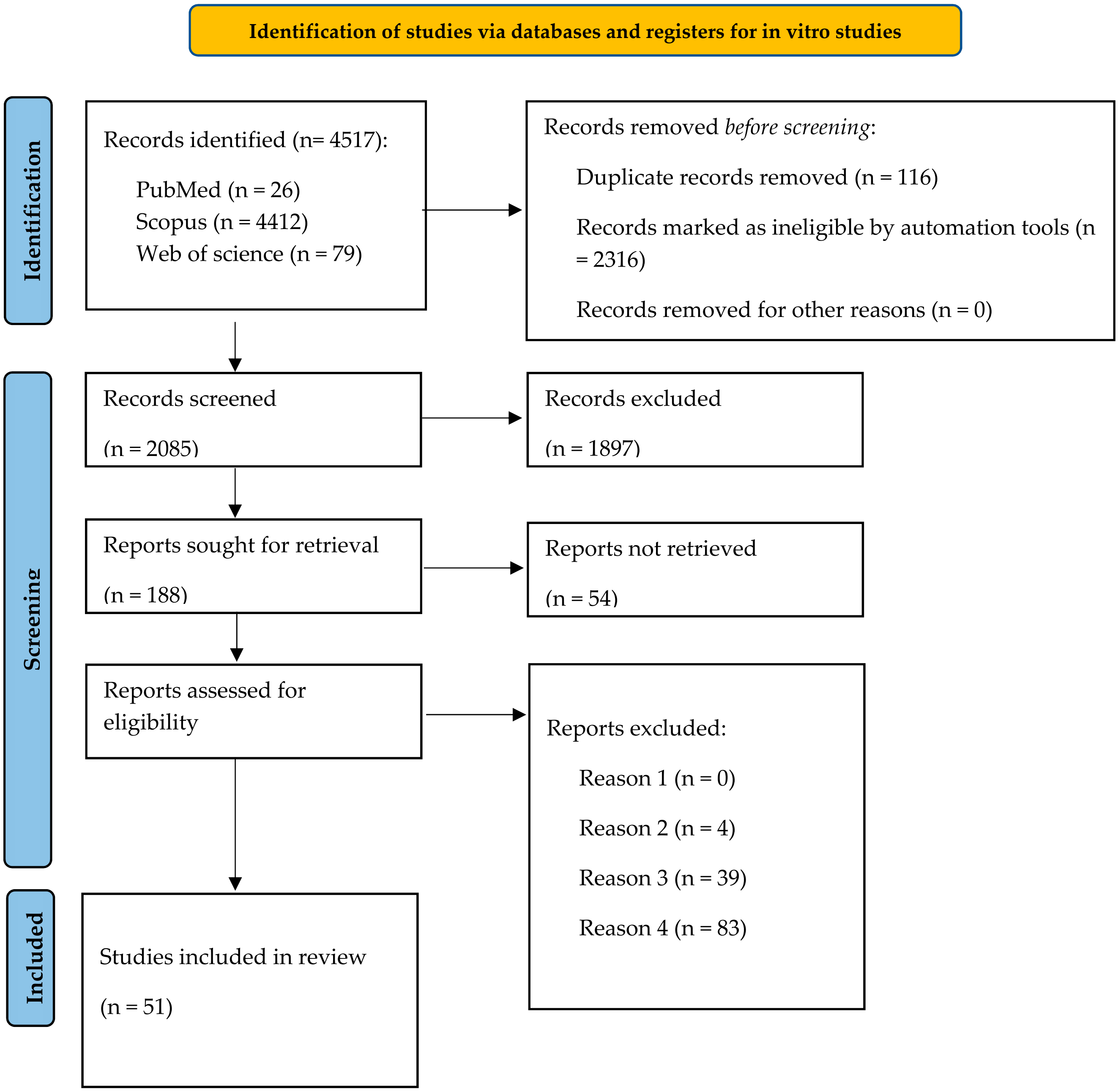

Exclusion criteria

- Reason 1: Studies and/or records published in a language different than English or Spanish
- Reason 2: Studies with pre-seeded cells
- Reason 3: Studies with insufficient data for retrieval
- Reason 4: Studies not relevant for the review

From: Page MJ, McKenzie JE, Bossuyt PM, Boutron I, Hoffmann TC, Mulrow CD, et al. The PRISMA 2020 statement: an updated guideline for reporting systematic reviews. BMJ 2021;372:n71. doi: 10.1136/bmj.n71

Supplementary data

Summary tables of descriptive analysis

Table S1. Summary of mechanical properties of TEGVs found in Clinicaltrials.gov

	POSS-PCU	Humacyte	Artegraft	BioIntegral Surgical No-React ®	TRUE	Saphenous vein	IMA
**Circumferential tensile strength (Kpa)**	No information	1400	1800	2500	3800	9760	4100
**Longitudinal tensile strength (Kpa)**	3210	1200	2500	10000	No information	2405	4300
**Suture resistance strength (Kpa)**	4460	2600	No information	No information	1950	3200	1350
**Dynamic compliance (%/100 mmHg)**	1.59	1.5	1.5	No information	No information	4.4	5.22

Table S2. Summary of mechanical properties of TEGVs in pre-clinical trials in animal models compared to saphenous vein grafts and internal mammary artery properties.

	PCL based TEVGs (n=26)	Decellularized TEGVs (n=4)	PLCL based TEGVs (n=5)	PU based TEVGs (n=6)	Saphenous vein	IMA
**Circumferential tensile strength (Kpa)**	2106 ± 1412	4350 ± 210	4233 ± 680	No data due to n=0	9760	4100
n of data analysed	10	2	3	0	-	-
**Longitudinal tensile strength (Kpa)**	3616 ± 1329	4600 ± 4942	9525 ± 6453	4212 ± 1789	2405	4300
n of data analysed	11	3	4	6	-	-
**Suture resistance strength (Kpa)**	1210 ± 920	6700 ± 7490	2600	2620 ± 1950	3200	1350

n of data analysed	7	2	1	3	-	-
**Dynamic compliance (%/100 mmHg)**	2.62 ± 1.95	No data due to n=0	8	2.62 ± 1.95	4.4	5.22
n of data analysed	5	0	1	2	-	-

Table S3. Summary of mechanical properties of TEGVs in in vitro studies compared to saphenous vein grafts and internal mammary artery properties.

	**PCL based TEVGs (n= 21)**	**TPU based TEVGs (n=5)**	**PLGA based TEVGs (n=4)**	**Saphenous vein**	**IMA**
**Circumferential tensile strength (Kpa)**	3594 ± 2486	2570	8350	9760	4100
n of data analysed	5	1	1	-	-
**Longitudinal tensile strength (Kpa)**	4107 ± 2376	15750 ± 4546	5008 ± 3468	2405	4300
n of data analysed	13	5	4	-	-
**Suture resistance strength (Kpa)**	4520 ± 4130	7860 ± 1600	1950 ± 1670	3200	1350
n of data analysed	3	2	2	-	-
**Dynamic compliance (%/100 mmHg)**	5.42 ± 2.65	No data due to n=0	3.41	4.4	5.22
n of data analysed	2	0	1	-	-

Tables from descriptive analysis of pre-clinical trials

Table S4. Primary materials used in the manufacture of TEVGs that have been tested on pre-clinical animal models.

**Primary materials used**	**Frequency**	**%**

PCL	26	46.43
SF	1	1.79
PHBV	2	3.57
PLCL	5	8.93
PGS	3	5.36
Decellularized Artery	4	7.14
ECM	1	1.79
PGA	1	1.79
PLLA	1	1.79
Chitosan	1	1.79
PU	6	10.71
CA	1	1.79
TEC	1	1.79
TPU	1	1.79
PLA	1	1.79
PLGA	1	1.79
Total	56	100

Table S5. Secondary materials used in the manufacture of TEVGs that have been tested on pre-clinical animal models.

**Secondary materials used**	**Frequency**	**%**
Chitosan	1	9.09
SF	1	9.09
Collagen I	3	27.27
Gelatin	2	18.18
PDO (Polydioxanone)	1	9.09
Biotin	1	9.09
PEGDA	1	9.09
Fibrin	1	9.09
Total	11	100

Table S6. Types of functionalization used in the manufacture of TEVGs that have been tested on pre-clinical animal models.

**Functionalization**	**Frequency**	**%**
Anticoagulant	10	38.46
Other peptides	3	11.54
Antibodies	1	3.85

Growing factors	4	15.38
Collagen	2	7.69
Antihyperlapsia	1	3.85
MMPs inhibitors	1	3.85
Antioxidant	1	3.85
Iloprost	1	3.85
CCL-2	1	3.85
Nitric Oxide release	1	3.85
Total	26	100
No functionalization	25 studies out of 43	53.4884

Table S7. Subgroup analysis of functionalization based on growing factors used in the manufacture of TEVGs that have been tested on pre-clinical animal models.

**Growing factors used for functionalization**	**Frequency**	**%**
VEGF	4	40
PDGR	1	10
bFGF	2	20
SDF 1a	3	30
Total	10	100

Table S8. Number of layers of TEVGs that have been tested on pre-clinical animal models.

**Vascular graft morphology**	**Frequency**	**%**
Monolayer	23	53.49
Bilayer	14	32.56
Tri-layer	3	6.98
No information retrieved	3	6.98
Total	43	100

Table S9. Integration technique for the development of TEVGs that have been tested on pre-clinical animal models.

**Integration made by**	**Frequency**	**%**
Blend	26	60.47
Coexists	4	9.3
No information retrieved	13	30.23
Total	43	100

Table S10. Primary manufacturing technique of TEVGs that have been tested on pre-clinical animal models.

Initial manufacturing technique	Frequency	%
Electrospinning	25	58.14
Freeze drying	3	6.98
Emulsion electrospinning	2	4.65
Pericardium decellularization	1	2.33
Spiral Shell implanted on dog	1	2.33
Felt	1	2.33
Wet Spinning	2	4.65
Phase Separation	1	2.33
3D printing	2	4.65
Aorta decellularization	3	6.98
Bio-tubing	1	2.33
Core coating	1	2.33
Total	43	100
Grafts requiring double manufacturing technique	8 out of 43	18.6
Monolayer	4	50
Bilayer	2	25
Tri-layer	2	25
Total	8	100

Table S11. Secondary manufacturing technique of TEVGs that have been tested on pre-clinical animal models.

Secondary manufacturing technique	Frequency	%
Electrospinning	3	37.5
Freeze thawing	1	12.5
Electrospinning yarn	2	25
Layer by layer assembly	1	12.5
Magnetic Field	1	12.5
Total	8	100
Not requiring second technique	35 out of 43	81.4

Table S12. Fiber diameter of TEVGs that have been tested on pre-clinical animal models.

Fiber diameter um (n= 31)	Values	
Mean	2.4	
Median	1	
SD	2.45	
95% CI	-0.05	4.85

Min - Max	0.18	8
No information retrieved	12 out of 43	27.91

Table S13. Internal diameter of TEVGs that have been tested on pre-clinical animal models.

Internal diameter mm (n= 42)	Values	
Mean	2.36	
Median	2	
SD	1.48	
95% CI	0.89	3.84
Min - Max	0.6	6
No information retrieved	1 out of 43	2.33

Table S14. Wall thickness of TEVGs that have been tested on pre-clinical animal models.

**Wall thickness mm (n= 41)**	**Values**	
Mean	0.49	
Median	0.5	
SD	0.34	
95% CI	0.15	0.83
Min - Max	0.11	2
No information retrieved	2 out of 43	4.65

Table S15. Animal models used in TEVGs that have been tested on pre-clinical animal models.

**Animal**	**Frequency**	**%**
Rat	28	65.12
Rabbit	3	6.98
Goat	1	2.33
Mice	2	4.65

Sheep	6	13.95
Pig	1	2.33
Dog	2	4.65
Total	43	100

Table S16. Vascular segments used for experimentation of TEVGs that have been tested on pre-clinical animal models.

**Vascular segment**	**Frequency**	**%**
Aorta	24	55.81
Carotid	13	30.23
Subcutaneous	4	9.3
Femoral	1	2.33
Arterial	1	2.33
Total	43	100

Table S17. Patency rates in months of TEVGs that have been tested on pre-clinical animal models.

**Patency months (n= 42)**	**Values**	
Mean	4.31	
Median	3	
SD	4.54	
95% CI	-0.23	8.85
Min - Max	0.1	18
No information retrieved	1 out of 43	2.33

Table S18. Presence of endothelization in TEVGs that have been tested on pre-clinical animal models.

**Endothelization**	**Frequency**	**%**
Yes	31	72.09
No	12	27.91
Total	43	100
No information retrieved	1 out of 43	2.33

Table S19. Cell infiltration rates of TEVGs that have been tested on pre-clinical animal models.

**Cell Infiltration % (n= 31)**	**Values**	
Mean	66.13%	
Median	70.00%	
SD	16.42%	
95% CI	49.71%	82.55%

Min - Max	30.00%	90.00%
No information retrieved	12 out of 43	27.91

Table S20. Porosity reported of TEVGs that have been tested on pre-clinical animal models.

Porosity % (n= 15)	Values	
Mean	78.36%	
Median	78.50%	
SD	11.64%	
95% CI	66.72%	89.99%
Min - Max	50.00%	91.60%
No information retrieved	28 out of 43	65.12

Table S21. Circumferential tensile strength of TEVGs that have been tested on pre-clinical animal models.

**Circumferential tensile strength MPa (n= 17)**	**Values**	
Mean	5.26	
Median	2.9	
SD	8.81	
95% CI	-3.55	14.07
Min - Max	0.03	35
No information retrieved	26 out of 43	60.47

Table S22. Longitudinal tensile strength of TEVGs that have been tested on pre-clinical animal models.

**Longitudinal tensile strength MPa (n= 31)**	**Values**	
Mean	7.63	
Median	4.74	
SD	7.62	
95% CI	0.01	15.24

Min - Max	0.3	35
No information retrieved	12 out of 43	27.91

Table S23. Burst strength of TEVGs that have been tested on pre-clinical animal models.

**Burst Strength mmHg (n= 24)**	**Values**	
Mean	1975.88	
Median	1600	
SD	1709.84	
95% CI	266.04	3685.71
Min - Max	356.9	8690
No information retrieved	21 out of 43	48.84

Table S24. Suture retention strength of TEVGs that have been tested on pre-clinical animal models.

**Suture retention strength N (n= 16)**	**Values**	
Mean	2.23	
Median	1.38	
SD	2.85	
95% CI	-0.63	5.08
Min - Max	0.17	12
No information retrieved	27 out of 43	62.79

Table S25. Kink diameter of TEVGs that have been tested on pre-clinical animal models.

**Kink diameter mm (n= 3)**	**Values**	
Mean	13.33	
Median	5	
SD	14.43	
95% CI	-1.1	27.77
Min - Max	5	30
No information retrieved	40 out of 43	93.02

Table S26. Dynamic compliance of TEVGs that have been tested on pre-clinical animal models.

**Dynamic compliance mmHg (n= 8)**	**Values**
Mean	73.88
Median	100
SD	59.16

95% CI	14.73	133.04
Min - Max	0.05	143
No information retrieved	35 out of 43	81.4

Tables from descriptive analysis of in vitro tested TEGVs

Table S27. Primary materials used in the manufacture of TEVGs that have been tested on in vitro models.

**Primary materials used**	**Frequency**	**%**
PCL	21	37.50
Chitosan	1	1.79
Collagen	2	3.57
Fibrinogen	1	1.79
HDMI-PCL-Arginine	1	1.79
Human Chorion	1	1.79
PBSU	1	1.79
PLCL	3	5.36
PU	1	1.79
PEA	1	1.79
PEUU	1	1.79
PGA	3	5.36
PGS	2	3.57
PLA	2	3.57
PLGA	4	7.14
TPU	5	8.93
POSS/PCUU	1	1.79
PTMCLLA	1	1.79
PU C45D	1	1.79
Decellularized ECM	1	1.79
PEO	1	1.79
PAN	1	1.79

Total	56	100.00

Table S28. Secondary materials used in the manufacture of TEVGs that have been tested on in vitro models.

Secondary materials used	Frequency	%
Collagen	6	23.08
Elastin	2	7.69
Gelatin	5	19.23
Alginate	2	7.69
DLPHEG	1	3.85
Chitosan	1	3.85
Carboxymethyl Chitosan	1	3.85
Silk Yarn	1	3.85
Egg shell	1	3.85
Matitol	1	3.85
Tropoelastin	1	3.85
Fibrinogen	1	3.85
Silk fibroin	1	3.85
Egg white-Sodium Alginate	1	3.85
Tecoflex (50%)	1	3.85
Total	26	100.00

Table S29. Types of functionalization used in the manufacture of TEVGs that have been tested on in vitro models.

Functionalization	Frequency	%
Anticoagulant	3	33.33
Collagen	6	66.67
Total	9	100.00

No functionalization	37 studies out of 46	80.43

Table S30. Number of layers of TEVGs that have been tested on in vitro models.

**Vascular graft morphology**	**Frequency**	**%**
Monolayer	25	54.35
Bilayer	11	23.91
Tri-layer	7	15.22
Multilayer (>3)	3	6.52
Total	46	100

Table S31. Integration technique for the development of TEVGs that have been tested on in vitro models.

**Integration made by**	**Frequency**	**%**
Blend	26	56.52
Coexists	20	43.48
Total	46	100

Table S32. Primary manufacturing technique of TEVGs that have been tested on in vitro models.

**Initial manufacturing technique**	**Frequency**	**%**
Decellularized	1	2.17
Dip Coating	3	6.52
Electrochemical	2	4.35
Electrospinning	29	63.04
Injected	3	6.52
Solvent Casting	6	13.04
Yarn braiding	2	4.35

Total	46	100

Table S33. Secondary manufacturing technique of TEVGs that have been tested on in vitro models.

**Secondary manufacturing technique**	**Frequency**	**%**
Spray-coating	1	14.29
Dip Coating	2	28.57
Electrospraying	1	14.29
3D Printing	2	28.57
Lyophilisation	1	14.29
Total	7	100
Not requiring second technique	39 out of 46	84.78

Table S34. Fiber diameter of TEVGs that have been tested on in vitro models.

**Fibber diameter um (n= 24)**	**Values**	
Mean	4.24	
Median	0.813	
SD	11.2109512	
95% CI	-6.97	15.45
Min - Max	0.16	54.6
No information retrieved	22 out of 46	47.82609

Table S35. Internal diameter of TEVGs that have been tested on in vitro models.

**Internal diameter mm (n= 35)**	**Values**
Mean	4.60

Median	4.50	
SD	2.90	
95% CI	1.70	7.51
Min - Max	0.7	15
No information retrieved	11 out of 46	23.91

Table S36. Wall thickness of TEVGs that have been tested on in vitro models.

**Wall thickness mm (n= 31)**	**Values**	
Mean	0.55	
Median	0.50	
SD	0.38	
95% CI	0.17	0.94
Min - Max	0.011	2
No information retrieved	15 out of 46	32.6087

Table S37. Porosity reported of TEVGs that have been tested on in vitro models.

**Porosity (n= 13)**	**Values**	
Mean	58.16	
Median	72.00	
SD	36.35	
95% CI	21.81	94.51
Min - Max	0.534	95.77
No information retrieved	33 out of 46	71.73913

Table S38. Circumferential tensile strength of TEVGs that have been tested on in vitro models.

**Circumferential tensile strength MPa (n= 13)**	**Values**
Mean	9.21

Median	5.39	
SD	16.49	
95% CI	-7.28	25.70
Min - Max	0.047	63
No information retrieved	33 out of 46	71.73913

Table S39. Longitudinal tensile strength of TEVGs that have been tested on in vitro models.

**Longitudinal tensile strength MPa (n= 37)**	**Values**	
Mean	94.30	
Median	4.57	
SD	508.29	
95% CI	-413.99	602.59
Min - Max	0.024	3100
No information retrieved	9 out of 46	19.56522

Table S40. Burst strength of TEVGs that have been tested on in vitro models.

**Burst Strength mmHg (n= 17)**	**Values**	
Mean	2161.14	
Median	1718.39	
SD	1835.61	
95% CI	325.53	3996.75
Min - Max	96.68	6262.93
No information retrieved	29 out of 46	63.04348

Table S41. Suture retention strength of TEVGs that have been tested on in vitro models.

**Suture retention strength N (n= 11)**	**Values**	
Mean	7.34	

Median	6.73	
SD	7.37	
95% CI	-0.04	14.71
Min - Max	0.43	27
No information retrieved	35 out of 46	54.34783

Table S42. Dynamic compliance of TEVGs that have been tested on in vitro models.

**Dynamic compliance mmHg (n= 6)**	**Values**	
Mean	327.28	
Median	349.50	
SD	179.50	
95% CI	147.78	506.79
Min - Max	110	540
No information retrieved	40 out of 46	54.34783
